# Cystatin C as a predictor of renal recovery and in hospital mortality in patients with acute kidney injury and liver cirrhosis

**DOI:** 10.1186/s12882-025-04341-7

**Published:** 2025-07-24

**Authors:** Eman Nagy, Ahmed H. Abdelfattah, Nagy Sayed-Ahmed, Sadiq Ahmed

**Affiliations:** 1 Mansoura Nephrology and Dialysis Unit, Internal Medicine Department, Faculty of Medicine, Mansoura University, Mansoura, Egypt; 2 Internal Medicine/Hospital Medicine, University of Kentucky College of Medicine, Lexington, KY USA; 3 Division of Nephrology Bone and Mineral Metabolism, University of Kentucky, Lexington, KY USA

**Keywords:** AKI, Liver cirrhosis, Mortality, Renal recovery, Cystatin C

## Abstract

**Background:**

Acute kidney injury (AKI) is a common and serious complication in patients with liver cirrhosis, with pre-renal AKI and acute tubular necrosis being the most frequent underlying causes. Cystatin C is a non-glycosylated 13 kDa protein that is consistently produced by all nucleated cells and has been suggested as a potential predictor of mortality in this patient population. The role of cystatin C in predicting renal recovery in these patients is not known and this was the aim of our study.

**Methods:**

This was a retrospective single center study that included hospitalized patients with liver cirrhosis who developed or were admitted with AKI and had serum cystatin C in their records from May 2017 to May 2023. The sociodemographic and laboratory data were retrieved from the data system. The in-hospital mortality, length of hospital stay, and renal recovery were recorded. Renal recovery was defined as a reduction in serum creatinine without needing dialysis on discharge.

**Results:**

This study included 209 patients with AKI and liver cirrhosis. Sixty-five patients (31%) died during hospital admission. The renal recovery was shown in 136 patients (65%). White blood cells, serum albumin, and peak serum cystatin C were the significant predictors for in-hospital mortality (*p* = 0.021, 0.013, and 0.001, respectively). Hypertension, serum albumin, baseline creatinine and baseline cystatin C were significant predictors of renal recovery in the studied patients (*p* = 0.017, 0.006, 0.030, and < 0.001, respectively). The cut-off value of baseline serum cystatin C for prediction of renal recovery was 2.62 with moderate sensitivity and specificity.

**Conclusion:**

In the current study, baseline serum cystatin C is a predictor of renal recovery in patients with AKI and liver cirrhosis. However, peak serum cystatin C is a predictor of mortality in these patients.

## Introduction

Acute kidney injury (AKI) is a life-threatening condition in hospitalized patients with liver cirrhosis. It is not an uncommon event in those patients, with an estimated prevalence of 20–50% [1]. Liver cirrhosis is a complex condition associated with significant morbidity due to enhanced vasodilation and large volume shifts. Therefore, kidney dysfunction adds another level of complexity to people who have cirrhosis [2]. Pre-renal AKI due to hypoalbuminemia, hepatorenal syndrome (HRS), and acute tubular necrosis are among the most common causes of AKI in patients with liver cirrhosis [1].

In addition to being a common condition, AKI is associated with prolonged hospital stays, poor patient outcomes, and high mortality [3, 4]. Moreover, it may lead to long-term impairment of kidney function [5].

Even though serum creatinine has been the gold standard diagnostic tool for kidney function for decades and is one of the variables used for calculating the Model for End-stage Liver Disease (MELD) score, it has several limitations. These limitations include its dependence on body weight, age, and gender [6]. Therefore, assessing kidney function using serum creatinine in patients with liver cirrhosis is inaccurate because of malnutrition associated with liver cirrhosis and decreased hepatic secretion of creatine [7].

Cystatin C, a nonglycosylated 13 kDa protein, belongs to the cystatin superfamily of cysteine protease inhibitors. All nucleated cells regularly produce cystatin C; it is then freely filtered at the glomerulus, primarily reabsorbed at the proximal tubule, and finally catabolized [8]. The level of serum cystatin C is not significantly impacted by age, gender, race, or muscle mass, in comparison to serum creatinine [9]. Serum cystatin C has been used as a biomarker for early diagnosis of AKI [10–12]. In addition, studies concluded that serum cystatin C might be an early predictor of AKI development in patients with liver cirrhosis [13–15]. Moreover, serum cystatin C may offer superior predictive capabilities compared to serum creatinine for assessing the outcome of AKI in patients with liver cirrhosis [15–17]. On the other hand, there is a scarcity of data about the predictive role of serum cystatin C in the recovery of AKI in patients with liver cirrhosis. Thus, the aim of the current study was to investigate the predictive efficacy of serum cystatin C for the recovery of kidney functions and mortality in hospitalized patients with liver cirrhosis and AKI.

## Methods

### Patients and setting

This retrospective cohort single-center study included all patients diagnosed with AKI and liver cirrhosis who were admitted to the University of Kentucky hospitals from May 2017 to May 2023. Adult patients over 18 years of age who met the criteria of liver cirrhosis and AKI during their admission and had serum creatinine and serum cystatin C levels measured within 7 days of admission in the electronic medical records, were included in the study. While, pregnant females, patients with malignancy, chronic kidney disease (CKD), thyroid dysfunction or advanced heart failure were excluded from the study. The study was adhered to the Declaration of Helsinki and was approved by the University of Kentucky institutional review board (IRB number 86422, dated 7/6/2024). Informed consent was waived due to the retrospective nature of the study and the minimal risk to participants.

### Data collection

The sociodemographic data, including age, gender, race, and smoking status, was collected. Medical history, including a history of diabetes mellitus and hypertension, was obtained. Laboratory parameters at day of admission, retrieved from the patients’ electronic records, included: white blood cells (WBCs, k/uL), blood hemoglobin (g/dL), platelets (k/uL), total protein (g/dL), serum albumin (g/dL), alanine transaminase (ALT, U/L), aspartate transaminase (AST, U/L), total bilirubin (mg/dL), prothrombin time (PT, seconds), international normalized ratio (INR), serum calcium (mg/dL), serum phosphorus (mg/dL), serum magnesium (mg/dL), total alkaline phosphatase (U/L), serum sodium (mmol/L), serum potassium (mmol/L), random blood glucose (mg/dL), serum creatinine (mg/dL), blood urea nitrogen (BUN, mg/dL), and serum cystatin C (mg/L). The peak serum creatinine refers to the highest serum creatinine for the patient during admission. The peak serum cystatin C corresponds to the serum cystatin C recorded on the same day as the peak serum creatinine. The baseline serum creatinine was defined as the first available value on admission. The need for renal replacement therapy (RRT) was defined as the presence of indications to initiate RRT, including severe hyperkalemia, metabolic acidosis, volume overload, or uremic complications such as uremic pericarditis or uremic encephalopathy [18].

### Diagnosis of AKI

Diagnosis of AKI was based on the criteria of the Kidney Disease Improving Global Outcomes criteria which defines AKI as a sudden decrease in renal function characterized by either an increase in serum creatinine of ≥ 0.3 mg/dL within 48 h or a ≥ 50% increase from baseline, or a reduction in urine output to < 0.5 ml/kg/h for 6–12 h without a previous history of kidney disease [19].

### MELD-Na score calculation

MELD-Na was used because it has been found to have a better fit for prediction of mortality in comparison to MELD score alone. It was calculated as follows:

MELD-Na = MELD Score - Na − 0.025 x MELD x (140-Na) + 140 [20].

### Outcome

The length of hospital stay, need for renal replacement therapy, occurrence of in-hospital mortality and renal recovery for the studied patients were collected from the patients’ electronic records. Complete renal recovery was identified when serum creatinine was returned to within 50% above its baseline. Partial renal recovery was defined when the patient was discharged without the need for renal replacement therapy but he did not meet the criteria of complete renal recovery [21].

### Statistical analysis

The Statistical Package for Social Science (SPSS) version 29 for Windows was used to analyze the collected data which were coded and processed using this program. Qualitative data were described as percentages and numbers, while quantitative data were described as means [± standard deviation (SD)] for parametric variables or medians (interquartile range; IQR), for nonparametric variables, as suitable. To assess the normality of distribution of variables, the Kolmogorov–Smirnov test was used. For comparing between two groups, t-test was used for normally distributed variables, while Mann Whitney test was used for non-normally distributed variables. The Chi-square test was used for comparing qualitative variables. Binary logistic regression analysis was used to recognize significant predictors of in-hospital mortality and renal recovery in the studied patients. Receiver operating characteristic (ROC) curve was performed to allocate a cut-off point of serum creatinine, serum cystatin C, and MELD-Na score to predict the occurrence of in-hospital mortality and renal recovery in these patients and the cut-off point was chosen relying on the best possible specificity without sacrificing the sensitivity of choice. The level of significance was considered at 5% (*P* ≤ 0.05).

## Results

The current study included 209 patients (112 males and 97 females) with liver cirrhosis who were admitted with or developed AKI during admission to the hospital. The mean age of the patients was 56 years. Eighty-six patients had diabetes mellitus, while 144 patients had hypertension. The median serum creatinine at admission was 1.71 mg/dL, while median peak serum creatinine was 2.72 mg/dL (IQR: 1.79–3.99). The median cystatin C at admission was 2.39 mg/L, while peak median cystatin C was 2.77 mg/L (IQR: 1.91–3.72). The median hospital stay was 12.97 days. Forty-nine patients (23.4%) needed RRT during the admission. Sociodemographic, clinical, baseline laboratory data of the patients were illustrated in Table 1.

**Table 1:** Basic characteristics of hospitalized patients with acute kidney injury and liver cirrhosis (*n* = 209)

Variable	Descriptive statistics
Age, years	56.16 ± 12.14 56(49.5–65)
**Gender**	
Male	112(53.6%)
Female	97(46.6%)
**Race**	
White	196(93.8%)
Black or African American	10(4.8%)
American Indian or Alaska Native	2(1%)
Filipino	1(0.5%)
**Hypertension**	144(63.9%)
**Diabetes Mellitus**	86(41.1%)
**Nicotine use**	65(31.1%)
**Laboratory data**	
White blood cells, k/uL	9.26(6.22–13.33)
Blood hemoglobin, g/dL	9.60(8.30–11.50)
Hematocrit, %	29.70(25.10–34.10)
Platelets, k/uL	111(68–176)
Total protein, g/dL	6.10(5.40–6.80)
Serum albumin, g/dL	2.90(2.40–3.50)
Alanine transaminase, U/L	29(17-51.50)
Aspartate transaminase, U/L	53(35–110)
Total bilirubin, mg/dL	2.40(1.03–5.98)
Prothrombin time, sec	19.30(16.53–24.98)
INR	1.70(1.30–2.30)
Serum calcium, mg/dL	8.55 ± 0.81
Serum phosphorus, mg/dL	3.90(3.10–4.98)
Serum magnesium, mg/dL	2(1.70–2.20)
Total alkaline phosphatase, U/L	145(98-207.5)
Serum sodium, mmol/L	134(129-138.5)
Serum potassium, mmol/L	4.20(3.70–4.80)
Serum chloride, mmol/L	100(94–105)
Random blood glucose, mg/dL	124(101-159.5)
Serum creatinine, mg/dL	1.71(1.24–2.71)
Blood urea nitrogen, mg/dL	39(24-66.75)
Serum cystatin C, mg/L	2.39(1.78–3.22)
**MELD-Na score**	26(18–31)

INR: International Normalized Ratio, MELD-Na: Model for End-stage Liver Disease-sodium

The data were expressed as mean ± SD, median (interquartile range), or number (%), as suitable

Sixty-five patients (31%) of patients died (non-survivor group), while 144 patients survived during admission (Survivor group). The patients with hypertension and diabetes were significantly more in the survivor group than the other group (73% vs. 58%, 46% vs. 29%, respectively). WBCs, ALT, AST, total bilirubin, prothrombin time, peak serum creatinine, peak serum cystatin C, and MELD-Na were significantly lower in the survivor compared to the non-survivor group. Conversely, serum albumin was significantly higher in the survivors than the non-survivors. Patients in the survivor group had longer hospital stays compared to those in the non-survivor group. Moreover, patients in the non-survivor group required RRT more frequently than those in the survivor group (Table 2).

**Table 2:** Comparison between survivors and non-survivors as regards different variables

	Survivors (*n* = 144)	Non-survivors (*n* = 65)	*p* value
Age, years	56.48 ± 11.94	55.45 ± 12.63	0.570
**Gender**			0.803
Male	78(54.2%)	34(52.3%)	
Female	66(45.8%)	31(47.7%)	
**Race**			0.566
White	133(92.4%)	63(96.9%)	
Black or African American	8(5.6%)	2(3.1%)	
American Indian or Alaska Native	2(1.4%)	0	
Filipino	1(0.7%)	0	
**Hypertension**	106(73.6%)	38(58.5%)	**0.029**
**Diabetes Mellitus**	67(46.5%)	19(29.2%)	**0.019**
**Nicotine use**	45(31.3%)	20(30.8%)	0.945
**Laboratory data**			
White blood cells, k/uL	8.57(6.04–12.42)	11.50(6.33–15.76)	**0.009**
Blood hemoglobin, g/dL	9.50(8.08–11.50)	10(8.50-11.65)	0.637
Hematocrit, %	28.90(24.78–34.23)	29.90(25.45–33.60)	0.962
Platelets, k/uL	103.50(66-191.25)	125(77.50–168)	0.890
Total protein, g/dL	6.15(5.50–6.90)	6.10(5.30–6.60)	0.149
Serum albumin, g/dL	3(2.50–3.60)	2.60(2.30-3)	**< 0.001**
Alanine transaminase, U/L	27(16.50–48)	34(22-65.50)	**0.030**
Aspartate transaminase, U/L	45(32–84)	78(46.50-145.50)	**< 0.001**
Total bilirubin, mg/dL	1.90(0.80–4.50)	3.30(1.55–8.85)	**0.013**
Prothrombin time, sec	18.60(16.20–24.60)	21.40(17.10–26.30)	**0.043**
INR	1.60(1.30–2.20)	1.90(1.40–2.40)	0.056
Serum calcium, mg/dL	8.61 ± 0.80	8.44 ± 0.82	0.157
Serum phosphorus, mg/dL	3.80(3.10–4.80)	4(3.15–5.95)	0.318
Serum magnesium, mg/dL	2(1.70–2.20)	2(1.70–2.20)	0.906
Total alkaline phosphatase, U/L	143(99.50–214)	157(93-206.5)	0.941
Serum sodium, mmol/L	134(130.25–139)	134(128–138)	0.596
Serum potassium, mmol/L	4.20(3.70–4.70)	4.30(3.55–4.85)	0.770
Serum chloride, mmol/L	101(95–106)	98(92.50–104)	0.259
Random blood glucose, mg/dL	126(106–164)	117(94.50-155.50)	0.145
Baseline serum creatinine, mg/dL	1.73(1.22–2.73)	1.70(1.29–2.51)	0.697
Peak serum creatinine, mg/dL	2.33(1.64–3.76)	2.97(2.31–4.23)	**0.004**
Blood urea nitrogen, mg/dL	38(22.25-61)	43(26.25-67)	0.478
Baseline serum cystatin C, mg/L	2.30(1.75–3.15)	2.63(1.89–3.30)	0.199
Peak serum cystatin C, mg/L	2.39(1.81–3.52)	3.28(2.80–4.51)	**0.001**
**MELD-Na score**	24(17–30)	27(20-33.5)	**0.019**
**Hospital stays**, days	13.83(7.08–25.92)	11.37(6.19–18.95)	**0.035**
**Need for renal replacement therapy**	22(15.3%)	27(41.5%)	**< 0.001**

INR: International Normalized Ratio, MELD-Na: Model for End-stage Liver Disease-sodium

The data were expressed as mean ± SD, median (interquartile range), or number (%), as suitable

The significant variables entered in a binary logistic regression equation to explore the most significant predictors of in-hospital mortality. It resulted in exclusion of hypertension, diabetes mellitus, AST, INR, MELD-Na score, and peak serum creatinine from being significant predictors of in-hospital mortality in the studied patients. While Peak serum cystatin C (OR = 2.808, *p* = < 0.001), followed by serum albumin (OR = 0.364, *p* = 0.013), and WBCs (OR = 1.085, *p* = 0.021) were the significant predictors of in-hospital mortality in the studied patients (Table 3).

**Table 3:** Binary logistic regression for in-hospital mortality

	Beta	OR	*p* value	95% confidence interval	
				Lower	Upper
Hypertension	-0.387	0.679	0.486	0.229	2.016
Diabetes mellitus	-0.653	0.520	0.268	0.164	1.652
White blood cells	0.081	1.085	**0.021**	1.012	1.162
Seum albumin	-1.011	0.364	**0.013**	0.163	0.811
Aspartate transaminase	0.000	1.000	0.586	0.999	1.001
INR	0.585	1.795	0.175	0.770	4.182
MELD-Na score	-0.072	0.931	0.136	0.847	1.023
Peak serum creatinine	-0.122	0.885	0.506	0.619	1.267
Peak serum cystatin C	1.033	2.808	**0.001**	1.517	5.199

INR: International Normalized Ratio, MELD-Na: Model for End-stage Liver Disease-sodium, OR: Odds Ratio

**Fig. 1:**
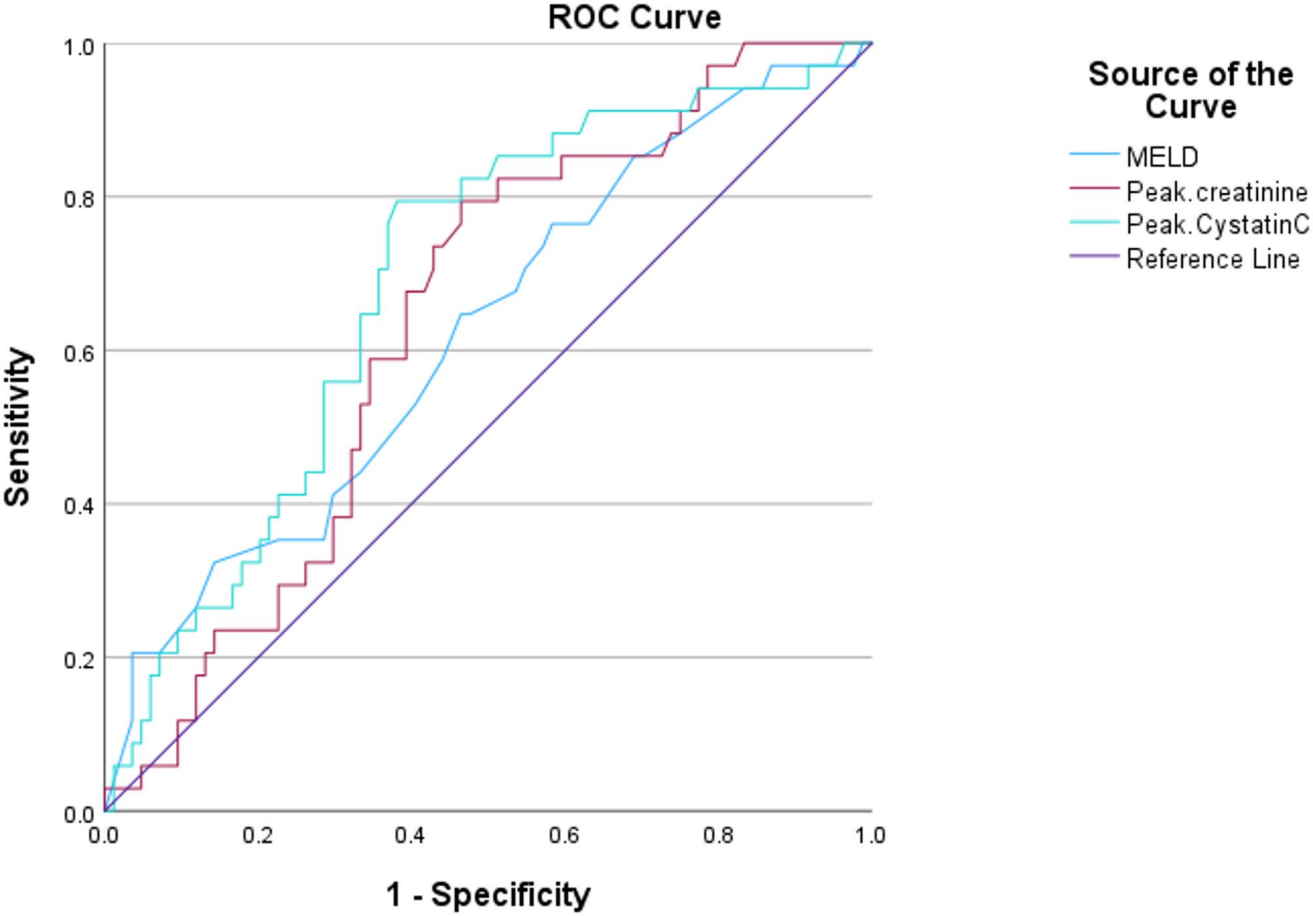
ROC curve for predictors of mortality

By constructing ROC analysis to test MELD-Na, peak serum creatinine, and peak serum cystatin C for prediction of in-hospital mortality, it showed that area under curves (AUCs) for prediction of in-hospital mortality using MELD-Na, peak serum creatinine, and peak serum cystatin C were 0.621, 0.638, 0.690, respectively. Using peak serum cystatin C optimal cut-off value of 2.77 mg/L, as determined by the Youden index, the corresponding sensitivity and specificity for prediction of in-hospital mortality were 79% and 62% respectively (Fig. 1; Table 4).

**Table 4:** ROC curve for predictors of mortality

	AUC	*p* value	Cut-off	Sensitivity	Specificity
MELD-Na	0.621	**0.040**	25.50	65%	54%
Peak serum creatinine	0.638	**0.019**	2.26	79%	54%
Peak serum cystatin C	0.690	**0.001**	2.77	79%	62%

AUC: area under the curve, MELD-Na: Model for End-stage Liver Disease-sodium, ROC: receiver operating characteristic

Seventy-three patients (34.9%) achieved complete recovery, while 63 patients (30.1%) experienced partial recovery (recovery group). Conversely, kidney function failed to recover in 73 patients (34.9%) (non-recovery group). Patients with hypertension were significantly more prevalent in the recovery group compared to the non-recovery group. WBCs, AST, peak serum creatinine, baseline and peak serum cystatin C levels, and MELD-Na score were significantly higher in the non-recovery group compared to the other group. On the contrary, serum albumin was significantly lower in the non-recovery group compared to the other group. Furthermore, patients in the non-recovery group required RRT more frequently compared to those in the recovery group (Table 5).

**Table 5:** Comparison between patients with complete/partial and non-renal recovery as regards different variables

	Non-recovery (*n* = 73)	Complete/Partial renal recovery (*n* = 136)	*p* value
Age, years	55.22 ± 12	56.66 ± 12.22	0.414
**Gender**			0.364
Male	36(49.3%)	76(55.9%)	
Female	37(50.7%)	60(44.1%)	
**Race**			0.434
White	71(97.3%)	125(91.9%)	
Black or African American	2(2.7%)	8(5.9%)	
American Indian or Alaska Native	0	2(1.5%)	
Filipino	0	1(0.7%)	
**Hypertension**	44(60.3%)	100(73.5%)	**0.048**
**Diabetes Mellitus**	24(32.9%)	62(45.6%)	0.075
**Nicotine use**	24(32.9%)	41(30.1%)	0.684
**Laboratory data**			
White blood cells, k/uL	11.04(6.33–15.46)	8.63(5.96–12.48)	**0.025**
Blood hemoglobin, g/dL	9.80(8.40–11.50)	9.55(8.20-11.53)	0.994
Hematocrit, %	29.80(25.05–33.60)	29.30(25.05–34.23)	0.779
Platelets, k/uL	125(73–170)	102(66–184)	0.683
Total protein, g/dL	6.10(5.30–6.60)	6.20(5.50–6.93)	0.191
Serum albumin, g/dL	2.70(2.30–3.20)	3(2.5–3.5)	**0.008**
Alanine transaminase, U/L	32(20–65)	28(16.5–49)	0.108
Aspartate transaminase, U/L	69(44.50–133)	45(32-86.5)	**< 0.001**
Total bilirubin, mg/dL	3.10(1.35–7.80)	2(0.9–4.6)	0.082
Prothrombin time, sec	20.95(17.05–25.78)	18.65(16.20–24.60)	0.096
INR	1.85(1.40–2.38)	1.60(1.30–2.20)	0.084
Serum calcium, mg/dL	8.48 ± 0.80	8.60(8.10–9.10)	0.244
Serum phosphorus, mg/dL	4(3.15-6)	3.80(3.10–4.70)	0.124
Serum magnesium, mg/dL	2(1.75–2.25)	2(1.7–2.2)	0.861
Total alkaline phosphatase, U/L	157(96.50–204)	143(98.5-215.5)	0.973
Serum sodium, mmol/L	134(128-139.50)	134(130–138)	0.916
Serum potassium, mmol/L	4.20(3.60–4.85)	4.20(3.70–4.70)	0.657
Serum chloride, mmol/L	98(93–104)	101(94–106)	0.295
Random blood glucose, mg/dL	117(98.5–156)	126(103–164)	0.272
Baseline serum creatinine, mg/dL	1.77(1.36–2.60)	1.69(1.16–2.72)	0.741
Peak serum creatinine, mg/dL	3.06(2.35–4.50)	2.28(1.61–3.47)	**< 0.001**
Blood urea nitrogen, mg/dL	44(26.25–67.75)	36.50(22.25-58)	0.230
Baseline serum cystatin C, mg/L	2.70(1.99–3.35)	2.24(1.72–3.10)	**0.028**
Peak Serum cystatin C, mg/L	3.31(2.82–4.64)	2.35(1.80–3.44)	**< 0.001**
MELD-Na score	27(19.50–33)	24(17–30)	**0.032**
Hospital stays, days	11.76(6.75–20.41)	13.15(6.93–25.58)	0.253
Need for renal replacement therapy	35(47.9%)	14(10.3%)	**< 0.001**

INR: International Normalized Ratio, MELD-Na: Model for End-stage Liver Disease-sodium

The data were expressed as mean ± SD, median (interquartile range), or number (%), as suitable

Binary logistic regression of renal recovery revealed that baseline serum cystatin C (OR = 0.446, *p* = < 0.001), hypertension (OR = 2.321, *p* = 0.017), serum albumin (OR = 1.953, *p* = 0.006), and baseline serum creatinine (OR = 1.415, *p* = 0.030) were the significant predictors of the renal recovery in the studied patients (Table 6).

**Table 6:** Binary logistic regression for renal recovery

	Beta	OR	*p* value	95% confidence interval	
				Lower	Upper
Hypertension	0.842	2.321	**0.017**	1.164	4.631
Seum albumin	0.669	1.953	**0.006**	1.212	3.144
MELD-Na score	0	1.000	0.911	0.999	1.001
Aspartate transaminase	-0.011	0.989	0.617	0.948	1.032
Baseline serum creatinine	0.347	1.415	**0.030**	1.035	1.933
Baseline serum cystatin C	-0.808	0.446	**< 0.001**	0.276	0.721

**Fig. 2:**
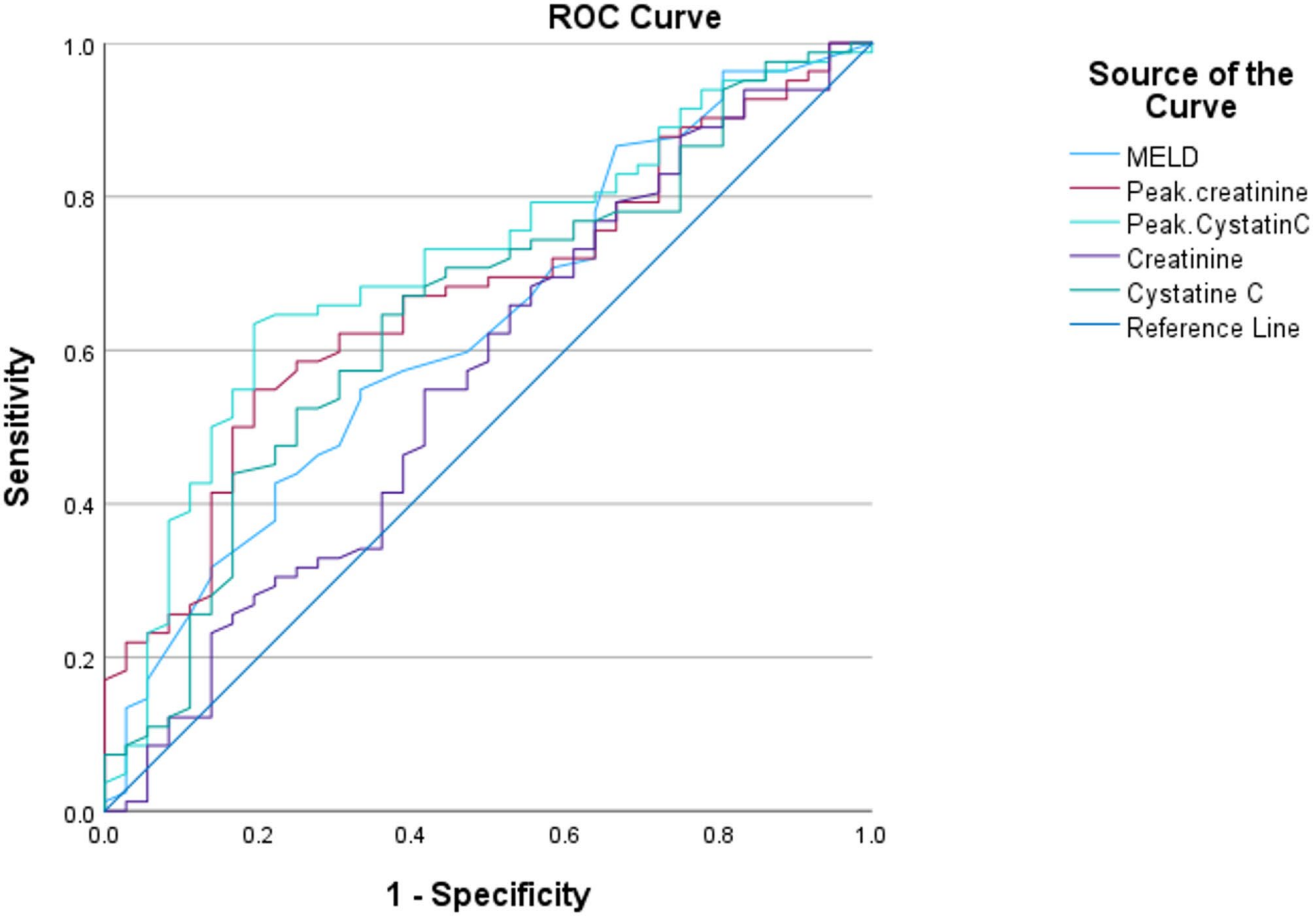
ROC curve for predictors of renal recovery

Through ROC analysis to assess MELD-Na, baseline, and peak serum creatinine levels, as well as baseline and peak serum cystatin C levels for predicting renal recovery, the corresponding AUCs were 0.631, 0.565, 0.671, 0.648, and 0.711, respectively. Utilizing a baseline serum cystatin C optimal cut-off value of 2.62 mg/L, as determined by the Youden index, yielded a sensitivity and specificity of 65% and 64%, respectively, for predicting renal recovery. Similarly, employing a peak serum cystatin C optimal cut-off value of 2.77 mg/L, determined by the Youden index, resulted in a sensitivity of 63% and specificity of 81% for predicting renal recovery (refer to Fig. 2; Table 7).

**Table 7:** ROC curve for predictors of renal recovery

MELD-Na	AUC	*p* value	Cut-off	Sensitivity	Specificity
	0.631	0.024	25.50	55%	67%
Baseline serum creatinine	0.565	0.262	1.89	55%	58%
Peak serum creatinine	0.671	**0.003**	2.26	55%	81%
Baseline serum cystatin C	0.648	**0.011**	2.62	65%	64%
Peak serum cystatin C	0.711	**< 0.001**	2.77	63%	81%

AUC: area under the curve, MELD-Na: Model for End-stage Liver Disease-sodium, ROC: receiver operating characteristic

## Discussion

This is a retrospective study that aimed to investigate the association between baseline serum creatinine and cystatin C levels with the outcomes in the form of in-hospital mortality and recovery of kidney functions in hospitalized patients with AKI and liver cirrhosis. It demonstrated that peak serum cystatin C was a significant predictor of in-hospital mortality in these patients. In addition, baseline serum cystatin C levels were identified to be a significant predictor of renal recovery.

Patients with hypertension were more common in the renal recovery group compared to the non-recovery group. This unexpected finding does not suggest that hypertension directly promotes recovery of kidney function in patients with AKI and liver cirrhosis. Rather, it may reflect other influencing factors such as patient characteristics or closer clinical monitoring among hypertensive patients. Further studies are warranted to explore the complex relationship between hypertension and AKI recovery.

Serum cystatin C level has been found to be a more sensitive marker for estimation of kidney functions and as an early predictor of kidney dysfunction [10, 22, 23]. Moreover, there is a growing body of research investigating its relationship with clinical outcomes. Several studies demonstrated a strong association between serum cystatin C and mortality in the general population reflecting proinflammatory and proatherogenic states [24–26]. Additionally, serum cystatin C was found to be significantly associated with mortality, regardless of kidney function, among elderly [27, 28], patients admitted to intensive care unit (ICU) [29, 30], human immunodeficiency virus (HIV)-infected persons [31, 32], and patients with cardiac diseases [33, 34],. This could be attributed to increased risk of hypertension, diabetes mellitus, myocardial infarction, heart failure, peripheral artery disease, and stroke with increasing levels of serum cystatin C [24]. In addition to the finding that serum Cystatin C levels demonstrate greater sensitivity in detecting early-stage kidney dysfunction compared to serum creatinine levels [35].

The occurrence of kidney dysfunction in cirrhotic patients is significantly associated with mortality [36]. In the current study, baseline serum cystatin C levels were not significantly associated with in hospital mortality in patients with liver cirrhosis. This could be because not all patients developed AKI upon hospital admission; instead, some developed AKI days after admission. Thus, peak serum cystatin C levels were used and identified as a significant predictor of in hospital mortality in patients with liver cirrhosis. Consistent with our findings, Maiwall et al. [37] found that serum cystatin C could accurately predict mortality in cirrhotic patients who developed AKI during their hospital admission. In addition, Aumpan et al. [38] demonstrated that serum cystatin C measurement within 24 h of admission could serve as a prognostic indicator for mortality in patients with decompensated cirrhosis. Furthermore, Hong et al. [39] concluded that redefining AKI using serum cystatin C levels could act as a predictor for in-hospital mortality among patients with liver cirrhosis with acute gastrointestinal bleeding. On the other hand, Abd El Wahab et al. [40] failed to find an association between mortality and serum cystatin C levels in patients with liver cirrhosis and AKI. This might be attributed to the older age of the cirrhotic patients in the latter study, as well as their higher baseline levels of creatinine and cystatin C compared to our patients.

Baseline serum cystatin C levels were found to be able to predict recovery of kidney functions in patients with AKI, particularly in subgroups like ICU and cancer patients. However, there is limited data on its predictive role in patients with liver cirrhosis. In the current study, higher serum cystatin C levels at admission were found to be a significant predictor of renal recovery in patients with liver cirrhosis. Serum cystatin was shown to be a predictor of dialysis requirements in ICU patients [41]. In addition, lower baseline serum cystatin C was linked to recovery of kidney functions in ICU patients [42]. Similarly, in patients admitted to coronary care unit, low serum cystatin C levels on the first day of admission were associated with renal recovery [43]. In the same way, higher baseline serum creatinine was associated with failure of renal recovery in patients with cancer who developed AKI [44]. Thus, this suggests that AKI patients with higher baseline serum cystatin C levels have a lower probability of resolution of AKI and restoration of their basal kidney functions. Thus, close monitoring with intensive treatment is necessary for these patients. More specifically, nephrotoxic drugs should be avoided, decreasing burden on kidneys should be adopted, and nephroprotective medications should be administered.

The limitations of the current study included the retrospective study design and its single-center nature. Additionally, the improvement of kidney function was defined based solely on serum creatinine levels, rather than considering urine output as well. However, it is recognized that urine output is not reliable criteria for monitoring AKI in these patients because of the inaccuracies in its collection in these patients. In addition, we did not have data on the causes of AKI or the underlying etiology of liver cirrhosis in our patients. As a result, we were unable to perform statistical analyses to explore their relationship with serum cystatin C levels or clinical outcomes.

In conclusion, this study demonstrates that peak serum cystatin C levels predict in-hospital mortality in hospitalized patients with AKI and liver cirrhosis. In addition, lower baseline serum cystatin C levels are associated with the recovery of kidney functions in these patients. Therefore, integrating serum cystatin C assessment into the management of AKI patients can help identify those with elevated levels who may benefit from targeted medical interventions to improve their outcomes.

## Data Availability

The data used is available from the corresponding author on reasonable request.
